# The Relationship between DNA Methylation and Antidepressant Medications: A Systematic Review

**DOI:** 10.3390/ijms21030826

**Published:** 2020-01-28

**Authors:** Lauren M. Webb, Kathryn E. Phillips, Man Choi Ho, Marin Veldic, Caren J. Blacker

**Affiliations:** 1 Mayo Clinic Alix School of Medicine, Rochester, MN 55905, USA; Webb.Lauren@mayo.edu (L.M.W.); Phillips.Kathryn@mayo.edu (K.E.P.); 2 Department of Psychiatry and Psychology, Mayo Clinic, Rochester, MN 55905, USABlacker.Caren@mayo.edu (C.J.B.)

**Keywords:** antidepressants, DNA, methylation, epigenetics

## Abstract

Major depressive disorder (MDD) is the leading cause of disability worldwide and is associated with high rates of suicide and medical comorbidities. Current antidepressant medications are suboptimal, as most MDD patients fail to achieve complete remission from symptoms. At present, clinicians are unable to predict which antidepressant is most effective for a particular patient, exposing patients to multiple medication trials and side effects. Since MDD’s etiology includes interactions between genes and environment, the epigenome is of interest for predictive utility and treatment monitoring. Epigenetic mechanisms of antidepressant medications are incompletely understood. Differences in epigenetic profiles may impact treatment response. A systematic literature search yielded 24 studies reporting the interaction between antidepressants and eight genes (*BDNF*, *MAOA*, *SLC6A2, SLC6A4*, *HTR1A*, *HTR1B*, *IL6, IL11*) and whole genome methylation. Methylation of certain sites within *BDNF*, *SLC6A4*, *HTR1A*, *HTR1B*, *IL11*, and the whole genome was predictive of antidepressant response. Comparing DNA methylation in patients during depressive episodes, during treatment, in remission, and after antidepressant cessation would help clarify the influence of antidepressant medications on DNA methylation. Individuals’ unique methylation profiles may be used clinically for personalization of antidepressant choice in the future.

## 1. Introduction

The World Health Organization (WHO) estimates 4.4% of the world’s population lives with depression [1]. Major depressive disorder (MDD) is a debilitating, heterogeneous disease that afflicts all sociodemographic backgrounds and cultures and is the leading cause of disability worldwide [2]. Depressive disorders are associated with higher rates of medical comorbidities [3,4] and increased risk of suicide. In 2015, suicide became one of the top twenty causes of death in the United States and was the second leading cause of death among 15–29-year-olds globally [1]. The economic burden of depression is high, costing the US $83.1 billion in 2000 [5]. Between 2005 and 2015, the total number of people living with depression increased by 18.4%, with a global population increase of approximately 12.8% during this period, only partly explaining this increase [1,6]. As the prevalence of MDD continues to rise, the WHO predicts that depression will be the leading cause of disease burden by 2030 [7].

Currently available treatments for MDD are often inadequate. Of patients treated for depression, 60–70% fail to achieve complete remission from symptoms [8]. For patients who display full or partial treatment response, most medications require weeks to months to take effect [9,10] and that may be after multiple medication trials, each with undesirable side effects and financial burden.

The etiology of MDD remains complex. Antidepressant medications still primarily target the monoamine system [11,12]. Genome-wide association studies of MDD in animals and humans have not fully explained the genesis of depression [13] or variable response to antidepressants [14]. For example, the discordance rate of MDD in monozygotic twins is heavily influenced by environmental factors [15]. Stressful life experiences, difficulties in role transition or functioning, and comorbid medical and psychiatric disorders have been associated with MDD across sociodemographic and cultural boundaries [16]. Since adverse life events do not always precipitate MDD, epigenetic mechanisms may impact the dynamic risk of depression [15].

Epigenetics describes modifiable, potentially heritable, non-nucleotide changes to DNA transcription [17,18,19]. These mechanisms can be both stable over time and dynamically responsive to environment. They can modify gene transcription/expression and organism physiology [20]. Epigenetics is crucial for normal neurogenesis and cell differentiation [9,10,11]. Experience-dependent DNA modulation provides one mechanism by which organisms and offspring can adapt to environmental changes [21]. Epigenetic responses occur following changes in nutrition, drugs, physical, and psychosocial factors [22].

Epigenetic mechanisms include post-translational histone modifications, noncoding RNAs, and DNA methylation [23]. Post-translational modifications to histone proteins (e.g., methylation, phosphorylation, acetylation) can increase or decrease DNA transcription [18,24]. Most RNA transcripts in humans do not encode functional proteins, such as microRNAs, piwiRNAs, endogenous small interfering RNAs, and long non-coding RNAs. On both the level of gene and chromosome, these noncoding RNAs are thought to regulate gene expression [25]. DNA methylation is the addition of a methyl to the cytosine pyrimidine ring of cytosine–phosphate–guanine dinucleotides (CpG) sites, which are especially numerous in the promoter regions [26,27,28]. DNA methylation typically suppresses gene expression by preventing transcription at the promoter [27,28,29] and by recruiting enzymes that promote suppression of gene expression to the chromatin. Thus, many genes display an inverse correlation between methylation level of the promoter and gene expression [20]. Although DNA methylation is known for its stability as a covalent modification [30], it is still responsive to environmental signals [28]. DNA methylation has been implicated in psychiatric disorders as a mechanism by which experience alters neuronal gene transcription [31], changing cognitive function and formation of long term memories [32] and emotional processing [33]. Altered DNA methylation and chromatin activation status has also been reported in human depression [26].

Because of the heterogeneity of MDD and typical onset of the disorder in adolescence or adulthood, the environment and DNA methylation likely play an important role in the pathogenesis and treatment of MDD [34,35]. Most antidepressant medications take weeks to months to exert therapeutic effects, despite higher monoamine levels in the brain occurring within days of initiating treatment. Antidepressant medications may interact with epigenetic mechanisms to create a “window of synaptic plasticity” [36]. One theory for the variability in response to antidepressants is the differential methylation of the CYP450 enzymes of the liver which metabolize antidepressant drugs, with altered enzyme expression possibly changing serum levels of the medications [37,38].

**Figure 1:**
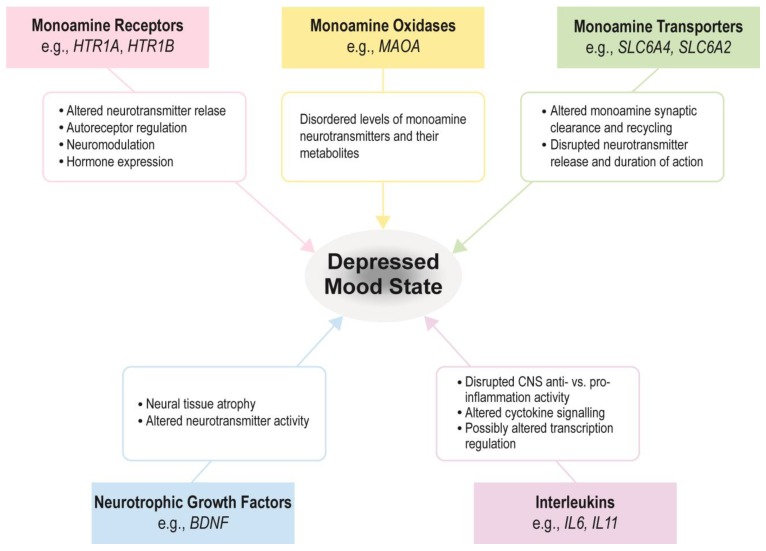
Plausible biologic mechanisms by which products of genes that have been studied in the context of antidepressants and DNA methylation contribute to depressed mood pathology.

In this systematic review, we review the literature studying the relationship between antidepressant use and DNA methylation of various genes in humans. Figure 1 illustrates how alterations in these genes’ products, which have been studied in the context of their DNA methylation, may contribute to the pathogenesis of depressed mood state. Examining the DNA methylation profiles of individuals prior to treatment with antidepressant medications may allow for prediction of efficacy and identification of the best medication for a particular patient. Understanding the epigenetic effects of medications may also help elucidate the pathophysiology of MDD.

## 2. Results

### 2.1. BDNF (Brain-Derived Neurotrophic Factor)

BDNF (brain-derived neurotrophic factor) promotes neuronal survival and synaptic plasticity [39] and is involved in learning, memory, and neurotransmitter release [40]. Animal studies show that *BDNF* is differentially expressed across brain regions and is highly influenced by environmental stressors such as foot shock and maternal deprivation [41,42]. Blaze et al. 2017 demonstrated increased DNA methylation in *BDNF* exon IV in the prefrontal cortices of prenatally stressed rats compared to non-stressed controls [43]. The neurotrophic hypothesis of depression suggests BDNF decreases in depression and that antidepressants increase *BDNF* expression [44]. Human studies have demonstrated decreased brain levels of BDNF in untreated MDD, and increased brain BDNF in those treated with antidepressants [45,46], possibly through alterations of DNA methylation. Table 1 summarizes the included studies of the relationship between *BDNF* DNA methylation and antidepressant medication use. 

Peripheral blood mononuclear cells (PBMC) have been used as a proxy for central nervous system (CNS) tissue. PBMC are non-invasive to collect from living subjects and serve as surrogates for brain tissue molecular changes, though there can be limited coherence between PBMC and CNS biomarkers in various circumstances [47,48]. D’Addario et al. 2012 examined antidepressant effects on *BDNF* DNA methylation using PBMC from forty-nine human subjects diagnosed with bipolar disorder type 1 (BD1), forty-five subjects with bipolar disorder type 2 (BD2), and fifty-two healthy controls (HC) [49]. Antidepressants elevated *BDNF* promoter methylation compared to controls. Antidepressant classes used included selective serotonin or norepinephrine reuptake inhibitors (SSRIs, SNRIs) or tricyclic antidepressants (TCAs). BD2 patients taking antidepressants combined with mood stabilizers had significantly increased *BDNF* promoter methylation and decreased *BDNF* gene expression compared with healthy controls and BD1. The same team then compared *BDNF* promoter methylation in forty-one MDD patients and forty-four controls [50]. MDD patients taking antidepressants plus mood stabilizers had lower *BDNF* promoter methylation compared to those treated with antidepressants alone. Regardless of current mood state, patients treated with SSRI or SNRI had increased *BDNF* promoter methylation relative to MDD patients taking SSRI/SNRI plus mood stabilizer. A 2014 study found that the *BDNF* promoter of exon I was hypermethylated in a group of 210 MDD compared to 60 BD (*p* = 0.0089) and 327 unaffected controls (*p* < 0.000) [51]. In support of D’Addario et al. 2012, the subset of 140 MDD patients treated with antidepressants had higher *BDNF* promoter methylation than the 25 MDD patients not treated with antidepressants (*p* = 0.0019) and to controls (*p* < 0.0001) [51]. These studies suggest divergent action of mood stabilizing and antidepressant drugs on *BDNF* promoter methylation.

Additional evidence that antidepressants increase *BDNF* promoter methylation in MDD came from an eight week trial of the SSRI, escitalopram, in eighty-five MDD patients [52]. Mean methylation across the entire *BDNF* gene was not associated with antidepressant response (*p* = 0.052), however, remitters had significantly increased *BDNF* promoter methylation at four specific amplicons of the *BDNF* promoter: 1, 3, 4, and 5. Patients with low baseline methylation levels had impaired response to escitalopram treatment. Eight weeks of escitalopram was associated with significantly increased average *BDNF* promoter and any amplicon methylation compared to pre-treatment in a subset of forty-four MDD subjects. In remitters, *BDNF* promoter methylation was significantly increased after eight weeks of escitalopram treatment, while in the non-remitter group, there was no significant increase in methylation post-treatment [52].

Although studies using PBMC demonstrated that antidepressants can induce *BDNF* promoter methylation [50,51], a 2015 study of buccal cells from 251 elderly patients with depression and 773 HC reported that chronic depression was associated with increased *BDNF* promoter methylation in elderly patients, but there were no significant interactions with antidepressants (SSRIs, TCAs, or other classes) [53].

Some studies have investigated the relationship between *BDNF* methylation and treatment response. In a naturalistic study of 108 patients with MDD, Kang et al. 2013 measured PBMC *BDNF* promoter methylation and suicidal ideation (SI) severity over a twelve week treatment period with one of nine antidepressants (amitriptyline, bupropion, escitalopram, fluoxetine, imipramine, mirtazapine, paroxetine, sertraline, and venlafaxine) chosen clinically [54]. Twenty-one subjects had a history of previous suicide attempt. There was no significant difference between the type of antidepressant and SI severity. When the patients were divided into high and low pre-treatment *BDNF* promoter methylation groups, lower methylation predicted significant improvement in SI severity. Higher *BDNF* promoter methylation was associated with male sex (*p* = 0.046), a history of suicide attempt (*p* = 0.035), and any suicidal ideation during antidepressant treatment (*p* = 0.013). Lower *BDNF* promoter methylation was associated with reduced SI following antidepressant treatment [54].

In contrast, another group found that hypomethylation of *BDNF* exon IV predicted poor response to antidepressant treatment [55]. They analyzed twelve CpG sites within *BDNF* exon IV in thirty-nine MDD patients. Pre-treatment methylation status at CpG position -87 predicted response to antidepressants. Patients without methylation at CpG-87 had a significantly higher risk for non-response than patients with methylation regardless of antidepressant class (SSRI, SNRI, mirtazapine, TCA, or monoamine oxidase inhibitor (MAO-I)). Patients with no methylation at CpG‑87 showed a decrease in plasma BDNF levels during the first week of antidepressant treatment, which has previously been predictive of nonresponse to antidepressants [56]. DNA methylation at the twelve CpG sites did not significantly change from baseline to the end of treatment [55].

In 2015 Kim et al. investigated *BDNF* gene methylation in acute coronary syndrome (ACS) patients with and without depression [57]. Of the 969 ACS patients, 378 had a depressive disorder. The depressed patients were randomized to receive a twenty-four week double-blind treatment with escitalopram + ACS treatment (*n* = 127), placebo + ACS treatment (*n* = 128), or ACS treatment alone (*n* = 123). Peripheral blood collected at baseline revealed that any depressive diagnosis was associated with higher *BDNF* methylation at CpG sites -1, -5, -8, and -9, and a higher average *BDNF* promoter methylation percentage. Those patients randomized to the escitalopram group had a higher remission rate (51.9%) than those in the placebo group (34.3%) (*p* = 0.011). When comparing depression remitters versus non-remitters, there was no significant difference in *BDNF* methylation percentages. However, within the escitalopram treatment group, a higher baseline *BDNF* exon VI methylation percentage was associated with higher rates of remission. In those patients in the placebo or medical treatment only groups, a higher *BDNF* methylation at CpG-1 of exon VI and a higher percent methylation of exon VI predicted persistence of depression at the one year follow-up. However, in the escitalopram group, there was no significant association between higher CpG-1 or global methylation and persistence of depression [57].

One study investigated the effect of antidepressants on methylation of H3K27 (histone H3, lysine 27) in male Canadians [58]. Trimethylation of H3K27 has been associated with repressed *BDNF* exon IV gene expression in a mouse model of depression [33]. Chen et al. 2011 measured *BDNF* exon IV expression and H3K27 trimethylation levels in post-mortem prefrontal cortex of MDD patients who had completed suicide with a history of, and toxicology positive for, antidepressants (*n* = 7), MDD subjects who completed suicide with no history of antidepressant use and negative antidepressant toxicology (*n* = 11), and control subjects with no psychiatric history (*n* = 9) [58]. Antidepressants taken by MDD patients included fluoxetine, venlafaxine, clomipramine, amitriptyline, citalopram, and doxepin. MDD subjects taking antidepressants had significantly lower H3K27 methylation than the MDD patients with no antidepressant treatment and controls. Additionally, antidepressants, regardless of medication class, increased *BDNF* IV expression above baseline levels. The study found no significant decrease in *BDNF* I, II, or III expression between MDD patients not treated with antidepressants and controls [58].

Lopez et al. 2013 also investigated H3K27 trimethylation of *BDNF* exon IV in a prospective study of twenty-five treatment-naïve MDD patients treated with citalopram for eight weeks [59]. Subjects were classified as responders or non-responders based on change in Hamilton Depression Rating Scale (HAM-D) score from baseline to eight weeks of treatment. Peripheral *BDNF* mRNA levels and *BDNF* exon IV H3K27 trimethylation were measured both at baseline and eight weeks of treatment. Responders exhibited a significantly increased peripheral *BNDF* mRNA levels after eight weeks on citalopram, while non-responders did not. The combined group of patients showed decreased H3K27 trimethylation after eight weeks of citalopram treatment (*p* < 0.001), although this was mostly explained by the responders. In the non-responder group, there was no significant difference in H3K27 trimethylation after treatment (*p* > 0.05). The responder group had significantly decreased H3K27 methylation after eight weeks of citalopram treatment compared to the non-responders, but there was no significant pre-treatment difference between the responders and non-responders (*p* > 0.05). Total peripheral BDNF and H3K27 trimethylation were significantly negatively correlated (r = −0.86; *p* < 0.0001). The study suggested that citalopram increases *BDNF* expression through alterations of H3K27 of the *BDNF* exon IV promoter [59].

### 2.2. MAOA

Monoamine oxidase A (MAO-A) is an enzyme responsible for metabolizing monoamine neurotransmitters, influencing the levels of monoamine neurotransmitters at the synapse [60]. It plays a role in mood, aggression [61], stress response [62], and brain development [63]. Table 2 summarizes the included studies of the relationship between *MAOA* DNA methylation and antidepressant medications. Checknita et al. 2018 examined methylation of the *MAOA* promoter in 114 young Swedish women with abuse histories compared to female controls [64]. Increased methylation of CpG-7/8 of *MAOA* occurred in women with active depression and lifetime use of psychotropic medication (stimulants, hypnotics, anxiolytics, antidepressants, and antipsychotics), but no significant differences in methylation occurred when lifetime antidepressant use alone was analyzed [64].

Domschke et al. 2015 hypothesized that hypomethylated *MAOA* in MDD may increase *MAOA* expression and thereby increase degradation of monoamines, potentially predicting reduced response to monoaminergic mechanisms of antidepressants [65]. Methylation of forty‑three CpG sites within the *MAOA* regulatory and exon I/intron I regions was investigated in ninety-four MDD patients treated with escitalopram. Treatment response was assessed by intra-individual differences in HAM‑D‑21 scores between the first and sixth week. In males, methylation of amplicons A and B was universally either absent or very low and had no significant association with treatment response. In females, average methylation across all CpG sites showed no significant association with clinical response to escitalopram. However, two individual CpG sites (CpG-1 in amplicon A and CpG-5 in amplicon B) did show nominal association with treatment response (*p* = 0.04 and *p* = 0.009, respectively). Lower methylation of these sites was associated with worse escitalopram response after six weeks. This trend was consistent with the group’s original hypothesis, predicting that higher levels of MAO‑A would lead to decreased efficacy of escitalopram [65].

### 2.3. SLC6A4

The serotonin transporter removes serotonin from the synapse to the presynaptic neuron for recycling, thus influencing the duration of action of serotonin at the synapse [66]. It is encoded by *SLC6A4* which has been implicated in risk of depression following emotional trauma. A study of thirty-three subjects with MDD and thirty-six HC investigated the associations between *SLC6A4* methylation and history of childhood abuse [67]. Increased methylation of the *SLC6A4* promoter at CpG-11 and -12 was associated with SSRI administration compared to no antidepressants (*p* = 0.02) or dual-action antidepressants (*p* = 0.009). After controlling for age, childhood trauma, and hippocampal volume, use of SSRI predicted a greater DNA methylation at CpG-11 and -12, suggesting SSRIs may increase DNA methylation at these sites, although sample size between antidepressant groups was very small [67].

DNA methylation in a CpG-rich region of the *SLC6A4* promoter was measured using peripheral blood samples of 108 Korean patients with MDD [68]. Methylation was measured before twelve weeks of naturalistic antidepressant treatment including amitriptyline, bupropion, escitalopram, fluoxetine, imipramine, mirtazapine, paroxetine, sertraline, and venlafaxine. Clinical outcomes were determined by comparison of baseline and post-treatment scores for depression, anxiety, social, and occupational functioning, disability, and quality of life. Lower average *SLC6A4* methylation and lower CpG-2 methylation were weakly associated with less depression improvement after antidepressant treatment. Increased CpG-1 methylation was associated with less improvement in anxiety and functioning. However, after Bonferroni correction, the strength of these associations was lost [68].

Domschke et al. 2014 investigated *SLC6A4* promoter methylation at nine CpG sites in PBMC from ninety-four MDD subjects treated with either escitalopram only (*n* = 61) or escitalopram and mirtazapine (*n* = 33). Forty subjects were co-medicated with antipsychotics and twenty-nine were co-medicated with mood stabilizers [69]. Clinical response was measured by inter-individual change in HAM‑D‑21 scores between the start and end of six weeks of escitalopram treatment. Patients with a lower average pre-treatment methylation of the nine CpG sites had impaired response to escitalopram compared to those patients with higher pre-treatment DNA methylation. Investigations of individual CpG sites, CpG-1 (*p* = 0.048), CpG‑2 (*p* = 0.002), and CpG‑4 (*p* = 0.029), showed (nominally) significant trends that low methylation at these sites was associated with decreased treatment response compared to subjects with higher baseline methylation [69].

*SLC6A4* promoter methylation was studied in twenty-eight MDD patients before and after eight weeks of antidepressant treatment and twenty-nine HC [70]. MDD patients were treated with a variety of antidepressants: Paroxetine, sertraline, escitalopram, mirtazapine, fluvoxamine, milnacipran, amitriptyline, and maprotiline. Pre-treatment, MDD patients had a significantly increased expression of the 5HTT protein compared to unaffected controls (*p* < 0.01) and expression levels significantly decreased post-treatment (*p* < 0.01). At baseline, decreased levels of methylation of CpG-3 and -5 were correlated with more severe depression symptoms (r = −0.41, *p* = 0.003; r = −0.38, *p* = 0.004). Lower baseline ClpG-2 methylation of the *SLC6A4* promoter was associated with increased clinical improvement in depression severity after eight weeks of treatment (r = −0.40; *p* = 0.04) [70].

Okada et al. 2014 measured DNA methylation rates in a CpG island of *SLC6A4* in the peripheral blood of fifty Japanese MDD patients before and after six weeks of antidepressant therapy with paroxetine, fluvoxamine, or milnacipran, and in fifty HC [71]. They were unable to distinguish between healthy controls and unmedicated MDD, or medicated/unmedicated MDD based on DNA methylation rates. Therapeutic response was defined based on improvement ratios (IRs), calculated by (HAM-D pre-treatment—HAM-D after six week treatment)/HAM-D pre-treatment. They discovered a significant increase in CpG-3 methylation after six weeks of antidepressant treatment. Furthermore, pre-treatment CpG-3 methylation rate was significantly, positively correlated with IR in MDD patients. There was no correlation between methylation change at CpG-3 between pre- and post-treatment and IR. Results suggested that higher pre-treatment CpG-3 methylation may predict better response to antidepressants [71].

### 2.4. SLC6A2

*SLC6A2* encodes a sodium:norepinephrine symporter responsible for presynaptically loading norepinephrine and maintaining norepinephrine homeostasis. Table 3 summarizes the studies relating DNA methylation of the neurotransmitter transporter genes and antidepressant medications. In MDD, panic disorder, and controls, there have been associations between *SLC6A2* promoter methylation and physiological factors such as BMI, heart rate, diastolic blood pressure, and age [72]. In a subset of five MDD and four panic disorder patients, two CpG sites (14 and 15 in Region A) were significantly hypermethylated following three month treatment with SSRIs (citalopram, fluoxetine, or sertraline) compared to pre-treatment, although the authors reported the methodology did not permit reliability of results [72].

### 2.5. HTR1A/1B

Serotonin receptor 5HT1A subtype variants have been associated with MDD and response to antidepressant treatment [73]. 5HT1A subtypes serve as serotonin autoreceptors and are involved in neuromodulation [74]. The 5HT1A receptor also has endocrine function, as agonism with ipsapirone promotes secretion of adrenocorticotrophin, cortisol, and growth hormone in humans [75]. 5HT1B has been associated with serotonin synthesis and influences behavior and anxiety in animal models [76]. Table 4 summarizes the studies of the relationship between DNA methylation of the serotonin receptor genes and antidepressant medications. After eight weeks of treatment with escitalopram in eighty-five Chinese Han MDD patients, analysis of a subset of 44 subjects showed that average methylation across *HTR1A* and *HTR1B* promoters did not significantly change [77]. However, four individual CpG sites demonstrated decreased methylation after treatment: *HTR1B*_1 amplicon CpG‑336, *HTR1B*_2 amplicon CpG‑105, *HTR1B*_2 amplicon CpG‑107, and *HTR1B*_4 amplicon 1443. They extended the analysis to patients who remitted following the eight week treatment and found that remitters had increased methylation at six other CpG sites within *HTR1A/1B* promoters (*HTR1A*_2 amplicon CpG‑2793, *HTR1A*_2 amplicon CpG‑2834, *HTR1A*_2 amplicon CpG‑2927, *HTR1A*_2 amplicon CpG‑2937, *HTR1B*_2 amplicon CpG‑100, *HTR1B*_4 amplicon CpG‑1401) [77].

*HTR1A* and *HTR1B* promoter methylation partially predicted response to antidepressant treatment. Average level of methylation of *HTR1A* or *HTR1B* in PBMC of depressed patients was not predictive of response to eight weeks of escitalopram treatment, but low pre-treatment methylation levels at CpG 668 (*p* = 0.025), and CpG‑1401 (*p* = 0.033) significantly predicted poor treatment response. An interaction was also discovered between Life Event Score (LES), methylation of four specific CpG sites (HTR1A_1 CpG‑659, CpG‑668, and CpG‑706, and HTR1B_2 CpG‑107), and response to escitalopram. Individuals with low LES scores and higher CpG methylation responded better to escitalopram than those with higher LES scores and lower DNA methylation [77].

Gassó et al. 2017 studied *HTR1B* promoter methylation in eighty-three child and adolescent subjects treated for 12 weeks with fluoxetine for MDD (*n* = 57), obsessive compulsive disorder (*n* = 16), and generalized anxiety disorder (*n* = 10) [78]. Lower *HTR1B* promoter methylation after treatment was associated with increased psychosocial functioning [78].

### 2.6. IL6 and IL11

Chronic inflammation of the CNS is associated with MDD [79] Altered levels of pro- and anti-inflammatory cytokine levels and the transcription factors that regulate them have been detected in patients with MDD [80]. The cytokine IL‑6 is increased in patients with MDD compared to healthy controls [81]. Table 5 summarizes studies of the relationship between DNA methylation of cytokine genes and antidepressant medication use. Ryan et al. 2017 compared methylation of the *IL6* gene in buccal cells from 92 >65-year-old subjects with and without MDD/high depressive symptoms and 288 HC [82]. Depression was associated with decreased *IL6* methylation compared to controls, while antidepressant use was associated with an increase. Over half of MDD subjects took antidepressants in the SSRI class [82]. Another study conversely observed decreased IL-6 levels after antidepressant treatment [83].

Results from the Genome-based Therapeutic Drugs for Depression Project (GENDEP) identified a single nucleotide polymorphism (SNP) in the cytokine *IL11* gene that predicted response to escitalopram [84]. *IL11* promoter methylation not only predicted response to antidepressants, but also to specific medications. Powell et al. 2013 studied DNA methylation of the *IL11* promoter in whole blood from 113 patients with moderate to severe MDD enrolled in GENDEP [85]. Patients were “partially randomized” to either twelve weeks of escitalopram (*n* = 80) or nortriptyline (*n* = 33) and baseline *IL11* promoter methylation was measured. Depression severity was scored weekly by Montgomery-Asberg Depression Rating Scale (MADRS). Lower baseline methylation levels of CpG‑5 predicted better response to both antidepressants (*p* = 0.005), while higher methylation of CpG‑4 was associated with better response to escitalopram, but lower response to nortriptyline (*p* = 0.005). Lastly, individuals homozygous for the G allele (GG) of the SNP rs1126757 had higher levels of methylation at CpG‑11 and better response to antidepressant treatment than those homozygous for the A allele (AA). There was no significant interaction between DNA methylation, the heterozygous genotype (AG), and response to antidepressants [85].

### 2.7. Global DNA Methylation

Takeuchi et al. 2017 examined genome-wide methylation in sixty-eight Japanese MDD patients treated with six weeks of paroxetine [86]. HAM-D-21 was used to assess psychological and physiological condition of patients at baseline, and weeks two, four, and six after paroxetine administration began. Two comparison groups were formed: The ten patients with the best response (BR) and worst response (WR) in HAM-D-21 scores. Comparing the BR and WR, HM450 DNA methylation analysis revealed 623 differentially methylated sites and 218 of them were nominally significantly different between BR and WR (*p* < 0.05). Two CpG sites were significantly differentially methylated: cg00594917 in the first exon *PPFIA4* which codes for liprin-α (*p* = 0.00012) and cg07260927 in the 5′ untranslated region of heparan sulfate-glucosamine 3-sulfotransferase 1 (*HS3ST1*) (*p* = 0.00013). Liprin proteins assist presynaptic neural transmission and interact with glutamate and muscarinic acetylcholine receptors. *HS3ST1* encodes the rate-limiting enzyme for heparan biosynthesis and has not previously been associated with the mechanism of action of paroxetine or neural signaling. Hierarchical cluster analysis within *PPFIA4* and *HS3ST1* distinguished most treatment responders. At all six CpGs of *PPFIA4*, the WR group was hypermethylated compared to the BR group. Similarly, five CpG sites in *HS3ST1* were differentially methylated between BR and WR [86].

Global DNA methylation patterns and methylation at certain sites correlate with biological age in humans [87]. This “epigenetic aging” was studied in 160 male combat-exposed veterans to investigate whether there was a difference in epigenetic age between subjects with and without post-traumatic stress disorder (PTSD) (*n* = 79; *n* = 81) [88]. Verhoeven et al. 2018 discovered that PTSD subjects counterintuitively had a younger epigenetic age than subjects without PTSD. Subjects taking antidepressants had a lower epigenetic age than those without antidepressants regardless of PTSD diagnosis (*p* = 0.017). Subjects with PTSD + antidepressant had reduced epigenetic age compared to healthy subjects. However, using the Sobel method, authors calculated that antidepressant use was not a statistically significant mediator of the association between change in epigenetic age and PTSD status. It remains unclear whether the association of antidepressants and lower epigenetic age was due to the medications themselves or differences in diagnosis severity, since patients who used antidepressants had higher rates of MDD and higher PTSD/depression scores (*p* < 0.001) [88]. Table 6 summarizes included studies relating global DNA methylation and use of antidepressant medications.

## 3. Discussion

Multiple studies have suggested that DNA methylation in humans can be altered by antidepressants and promote mood symptom remission. Additionally, pre-treatment DNA methylation profiles of certain genes may predict an individual’s likelihood of achieving remission, allowing clinicians to personalize and optimize treatment. The interaction between DNA methylation and antidepressants has been studied in cell lines, animals, and humans via global DNA methylation and specific genes.

The epigenetic effects of antidepressants on the *BDNF* gene are the best studied, but the results remain difficult to interpret. The neurotrophic hypothesis of depression posits that depression should be associated with decreased *BDNF* expression [89], potentially driven by *BDNF* hypermethylation. However, four studies reported antidepressant use associated with hypermethylation of the *BDNF* promoter [49,50,51,52]. D’Addario et al.’s 2012 study explored bipolar subtypes and found higher methylation in BD2, a subtype which may present with more depressive episodes than BD1, although this was not explicitly explored in the paper [49]. However, the finding that higher levels of DNA methylation were observed in subjects on pharmacological treatment with mood stabilizers plus antidepressants compared to mood stabilizers alone is more complicated; it may reflect antidepressants being used in subjects with more frequent depressive episodes, increased depressive severity, or treatment resistance. More confusing was the Carlberg et al. 2014 finding that hypermethylation was significantly associated with antidepressant therapy but not depression severity [51]. To tease apart these questions would require a study of *BDNF* methylation of subjects with acute and chronic depressive episodes, at baseline, during treatment, after depression remission, and after antidepressant cessation.

Studies have shown that early increases in serum and plasma *BDNF* levels within the first seven to fourteen days of starting an antidepressant medication may predict improvement in MDD symptoms after six weeks of treatment [56,90]. Counterintuitively, a pre-treatment hypomethylated *BDNF* promoter has been associated with poor antidepressant response [52,55]. Perhaps antidepressants increase *BDNF* expression through a mechanism other than decreasing methylation of the *BDNF* promoter, such as by decreasing H3K27 methylation in *BDNF* exon IV. Studies of H3K27 methylation of the *BDNF* exon IV promoter highlight the consistency of methylation changes across PBMC and postmortem prefrontal cortex samples. Lopez et al. 2013 confirmed that antidepressant treatment decreased H3K27 methylation in PBMC [59], as Chen et al. 2011 also demonstrated decreased H3K27 methylation in postmortem prefrontal cortex samples from MDD subjects who were taking antidepressants [58]. Antidepressants’ effects on histone modifications may be more important in increasing *BDNF* expression than DNA methylation of the promoter. *BDNF* promoter hypomethylation predicted poor response to antidepressant treatment [52,55], but corresponded with greater improvement in suicidality in a separate study [54]. However, increased *BDNF* promoter methylation after antidepressant treatment correlated with depression remission [52,57].

Kim et al. 2015 hypothesized that antidepressants may be more active on *BDNF* that is hypermethylated because *BDNF* methylation may be required for binding of methyl-CpG-binding protein 2 (MECP2) [57]. One of the proposed mechanisms for antidepressant action increasing *BDNF* expression is through phosphorylation of MECP2 and subsequent dissociation of MECP2 from DNA. Kim et al. 2015 found that depression persistence was associated with higher baseline *BDNF* methylation only in the placebo or medical treatment only group but not in the escitalopram group. They postulated that escitalopram caused MECP2 to dissociate from DNA, preventing depression persistence [57]. Tadić et al. 2014 made the same argument about the requirement of DNA methylation for MECP2 binding regarding their observation that lack of methylation at CpG-87 in the *BDNF* exon IV promoter was associated with non-response to treatment and a decrease in peripheral *BDNF* [55].

Analysis of buccal-derived DNA reported that MDD in elderly patients was associated with hypermethylated *BDNF* promoters, with no effect of antidepressant treatment [53]. This finding of hypermethylated *BDNF* is consistent with most studies of methylation status of *BDNF* in MDD or other depressive disorders [91]. Whether this reflected treatment-resistance, severity, or an age-related effect is unclear. It is also important to consider that different tissues as well as different regions within the CNS have different methylation profiles [92]. Which better reflects the methylation status of the CNS with regard to *BDNF* methylation remains to be discovered because the blood and buccal tissue types are almost equally concordant with the brain [93].

In light of the monoamine hypothesis of depression, investigators have studied *MAOA* DNA methylation and its interaction with antidepressants. The enzyme product of *MAOA* breaks down the same monoamines increased at the synapse by SSRIs. Checknita et al. 2018 did not find a significant effect of antidepressants on *MAOA* methylation in saliva, but it is unclear how well the DNA methylation in heterogeneous saliva cell samples correlates to cells of the CNS [64]. Domschke et al. 2015 found a nominal association between 2 CpG sites within *MAOA* and response to escitalopram [65]. They discovered that hypomethylation of *MAOA* led to worse response to antidepressant medications. They hypothesized that this was due to increased MAO-A available to counteract the effect of SSRIs. Interestingly, *MAOA* often fails to escape X-inactivation, putting females at increased risk of excessively high MAO-A levels and consequently deficient levels of serotonin and norepinephrine. These two studies alone do not provide enough evidence that *MAOA* promoter methylation plays an important role in antidepressant action or treatment response. Future studies could consider measuring MAO-A protein levels in patients with hyper- and hypo-methylated *MAOA* and measure response to an MAO-I class antidepressant [65]. One obstacle to this type of study is that MAO-I antidepressants are rarely used clinically due to their side effect and safety profile, and animal models may be required instead.

Investigators also studied *SLC6A4*, a serotonin transporter responsible for removal of serotonin from the synapse, as decreased expression of this gene could lead to more serotonin in the synapse, but less serotonin recycling. Booij et al. 2015 discovered that SSRIs increase promoter methylation at CpG-11 and -12 when compared to no antidepressants or non-SSRI antidepressants [67]. This suggests that the methylation of the *SLC6A4* gene may be a mechanism unique to SSRIs. Okada et al. 2014 discovered a significant increase in CpG-3 methylation after six weeks of treatment with either SSRI or SNRI [71]. Domschke et al. 2014 discovered a significant association between low promoter *SLC6A4* methylation and impaired response to escitalopram [69]. Similarly, Iga et al. 2016 discovered that decreased levels of CpG-3 and CpG-5 methylation were associated with more severe depression and increased CpG-2 methylation was associated with increased clinical improvement with treatment [70]. Higher pre-treatment methylation of CpG-3 likewise correlated with increased improvement in clinical symptoms of depression [71]. Based on these studies, *SLC6A4* methylation appears to be increased by antidepressants and increased promoter methylation is predictive of better treatment response.

Methylation status of specific CpG sites within *HTR1A* and *HTR1B* predicted treatment response, as shown by Wang et al.’s 2018 finding that methylation of certain CpG sites only increased in the subjects who remitted from their depression [77]. This may reveal one of the mechanisms of action of escitalopram in treating depressive symptoms, potentially decreasing expression of these serotonin transporters and potentiating serotonin in the synapse. Gassó et al. 2017 found that lower pre-treatment average *HTR1B* promoter methylation is associated with increased patient functioning after fluoxetine treatment [78]. Their hypothesis was that patients with lower pre-treatment methylation had high *HTR1B* expression and lower serotonin, so they were able to gain more clinical improvement from fluoxetine than those with lower initial *HTR1B* expression.

Studies of cytokines have also provided insight into potential mechanisms of antidepressants in reducing inflammation. For example, Ryan et al. 2017 showed an increase in methylation of the *IL6* promoter with antidepressant use which could presumably lower IL-6 levels [82]. Additionally, Powell et al. 2013 presented how differential methylation of the *IL11* gene could distinguish between better treatment options for individuals: Subjects with hypermethylated CpG-4 responded better to escitalopram, while those with hypomethylation responded better to nortriptyline [85]. IL-11, along with other cytokines like TNF-α and IL-6, has been shown to interfere with serotonin signaling [94]. These studies implicate cytokines as potential targets for novel antidepressants. Differences in DNA methylation at certain CpG sites can serve as guides for clinicians to choose the optimal medication.

Examining the effect of antidepressants on the global methylome yielded discoveries of two genes that are differentially methylated in patients who were the worst and best responders to paroxetine [86]. Takeuchi et al. 2017 identified *PPFIA4* and *HSG3ST1* as genes of future investigation related to antidepressant mechanism and response. The methylation status of certain CpG sites within these genes could explain individual variability in response to paroxetine treatment [86]. Verhoeven et al.’s 2018 study of veterans with and without PTSD introduced the idea that antidepressant medications may have a global effect on DNA methylation patterns that alter one’s epigenetic age [88]. The long-term effects of these medications on not only depression but general medical health are yet to be explored [88].

The results of studies investigating the relationship between antidepressant medications and DNA methylation can be difficult to compare due to the different genes, tissues, and CpG sites examined in each experiment. There are countless approaches to measure subjects’ response to antidepressants, including scales of depression severity, functional level, suicidality, residual symptoms, and number of failed antidepressant trials [86]. Furthermore, the articles in our review often studied distinct populations that prevent generalizability of the results, such as combat-exposed male veterans [88] or females who had experienced physical and/or sexual abuse [64]. Methylation profiles of these samples may be affected by life experiences, comorbid anxiety disorders [65], or age [95]. As these were all studies of human subjects, researchers could not ensure medication compliance or select patients who were taking a single class of antidepressant medication [58]. Because many of these were naturalistic studies, there was no way to standardize antidepressant dosing regimens or confirm whether antidepressant dose affects DNA methylation [52].

Further investigation into the relationship between antidepressants and DNA methylation is warranted, as these preliminary studies used relatively small sample sizes. Researchers have been able to detect CpG sites significantly associated with antidepressant response or non-response, but it is still unclear whether these differences are biologically and clinically meaningful [85]. Although blood, buccal cell, and saliva have been accepted as reasonable surrogates for DNA methylation in the brain, future studies should also explore the effect of antidepressants on DNA methylation in the brain [49,51]. It will be important to conduct randomized, controlled trials to distinguish the effect of pharmacologic treatment on DNA methylation from the effect of the MDD phenotype itself [51]. As MDD can be a chronic condition, there are also opportunities to study the change in DNA methylation longitudinally over years in genes such as *BDNF* or *IL6* and investigate whether DNA methylation is dynamically linked to incidence, severity, and remission from MDD [53,82]. Furthermore, analysis of global DNA methylation has already implicated genes like *HSG3TS1* that were not previously associated with neural signaling as potential players in MDD pathogenesis and antidepressant response [86].

## 4. Materials and Methods

**Figure 2:**
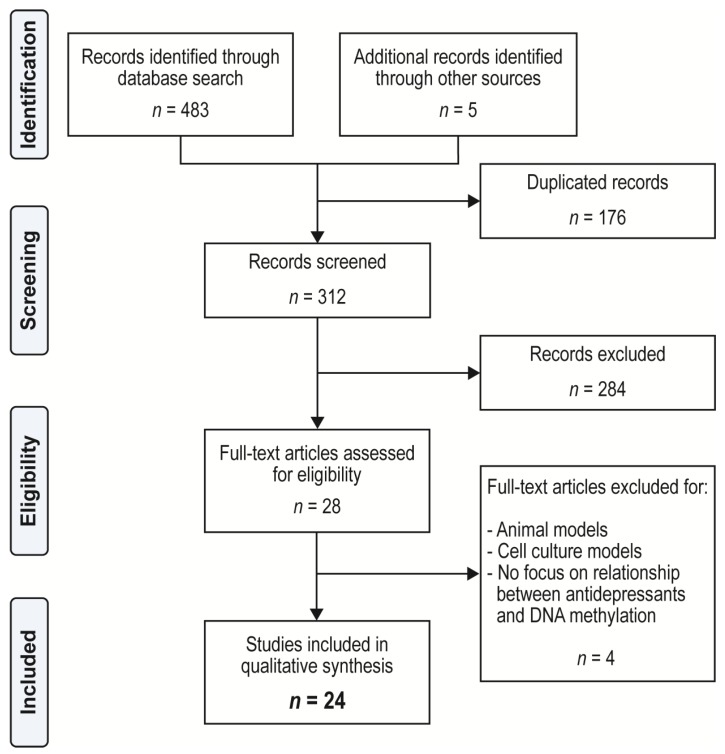
Preferred Reporting Items for Systematic Reviews and Meta-Analyses (PRISMA) flow diagram for systematic literature review [99]. Twenty-four articles met full inclusion criteria. The articles described the relationship between antidepressant administration and DNA methylation in the following genes: Brain-derived neurotrophic factor (*BDNF*) [49,50,51,52,53,54,55,57,58,59] (Table 1), monoamine oxidase A (*MAOA*) [64,65] (Table 2), 5-hydroxytryptamine (serotonin) transporter (*SLC6A4*) [67,68,69,70,71] (Table 3), norepinephrine transporter (*SLC6A2*) [72] (Table 3), 5-hydroxytryptamine transporters 1A and 1B (*5HTR1A/1B*) [77,78] (Table 4), interleukin-6 (*IL6*) [82], and interleukin-11 (*IL11*) [85] (Table 5). Two papers reported antidepressant effects on global DNA methylation [86,88] (Table 6).

We conducted a comprehensive literature search of PubMed, PsycINFO, and SCOPUS from 24 January 1975 (discovery of transcriptional control by DNA methylation [96]) to 22 June 2019. Database-specific truncations were used. The search string was Title/Abstract/Keywords (“antidepressant” OR “anti-depressant” OR “SSRI” OR “selective serotonin reuptake inhibitor” OR “SNRI” OR “selective norepinephrine reuptake inhibitor” OR “MAO*” OR “bupropion” OR “tricyclic antidepressant” OR “TCA”) AND (“epigen*”) AND (“methylat*”). Articles were collated in Endnote X9 (Clarivate Analytics, Philadelphia, PA). Duplicates were removed manually by the first author. Each abstract was reviewed independently by the first and second authors. Twenty-eight articles were selected for full text review (Figure 2). Inclusion criteria were: (1) Discussed the effect of antidepressant medications on DNA methylation in humans or the ability of DNA methylation to predict clinical response to antidepressant treatment in humans; (2) and full-text was available in English. Exclusion criteria were: (1) Studies that examined the effects of antidepressants on DNA methylation in utero (reviewed in Gentile and Fusco 2017 [97]). Each experimental paper was reviewed for design, sample size, description and duration of experiment, types of controls, collection of results, analysis, and findings. Data were extracted independently and in duplicate by the first and second authors. Studies were appraised for quality and risk of bias using a modified published process [98].

## 5. Conclusions

These studies demonstrate that epigenetic profiles have the potential to be used clinically to make decisions regarding antidepressant therapy. Choosing the best medication for a patient’s unique epigenome could optimize treatment effectiveness and reduces the time spent suffering. Epigenomic profiling may eventually be undertaken routinely with pharmacogenomics as part of individualized treatment plans. However, translating the biochemical data into clinical guidelines will require further investigation of different antidepressant class effects on epigenetic signaling, and their correlation with treatment response in various disease phenotypes.

## Figures and Tables

**Table 1:** Summaries of studies exploring relationship of antidepressants and *BDNF* DNA methylation.

Reference	Genomic Region Studied	Study Description	Tissue Examined	Findings
D’Addario et al., 2012 [49]	Exon I promoter (chr 11: 27 743 605–27 744 379)	Milanese study of *BDNF* methylation using fluorescence-based RT-PCR^1^ in bipolar disorder (BD) patients on mood stabilizers, BD patients on antidepressants + mood stabilizers, and healthy controls.	Peripheral blood mononuclear cells (PBMC)	● *BDNF* promoter methylation was increased in BD2 compared to controls, but not in BD1 compared to controls. ● *BDNF* promoter methylation was increased in patients using antidepressants compared to controls and patients taking mood stabilizers alone.
D’Addario et al., 2013 [50]	Exon I promoter	Milanese study of *BDNF* methylation using fluorescence-based RT-PCR in major depressive disorder (MDD) on antidepressants, MDD on antidepressants + mood stabilizer, and healthy controls.	PBMC	● MDD patients treated with antidepressants (serotonin or norepinephrine reuptake inhibitors (SSRIs or SNRIs)) alone had higher *BDNF* promoter methylation compared with patients receiving antidepressant + mood stabilizer.
Carlberg et al., 2014 [51]	Exon I promoter	Austrian study of *BDNF* methylation using PCR on bisulfite-converted genomic DNA from white, European MDD, BD, and unaffected controls. Subgroup of MDD patients analyzed for effects of antidepressants on DNA methylation.	PBMC	● MDD subgroup treated with antidepressants had significantly increased *BDNF* promoter methylation compared to controls and MDD patients not treated with antidepressants. ● No increase in % of methylated reference values in MDD without antidepressant therapy compared to control subjects.
Wang et al., 2018 [52]	Five CpG islands within the promoter.	Measured methylation of *BDNF* via PCR amplification of bisulfate-converted DNA in Han Chinese MDD patients before and after 8 weeks of 10–20mg escitalopram daily.	Whole blood genomic DNA isolate	● Methylation of 4 amplicons in *BDNF* (1, 3, 4, and 5) was significantly associated with response to escitalopram after 8 weeks, with higher methylation status associated with better response to escitalopram ● Patients with lower LES^2^ and higher DNA methylation responded better to escitalopram than those with higher LES and lower methylation. ● After 8 weeks of escitalopram, average *BDNF* and any *BDNF* amplicon methylation were significantly increased compared to baseline. ● In the remitter group, escitalopram treatment significantly increased DNA methylation, while in the non-remitter group there was no significant increase in *BDNF* methylation.
Januar et al., 2015 [53]	Exon I and IV promoters	Bisulfite conversion and PCR measurement of *BDNF* methylation in >65-year-old French patients with or without depression.	Buccal swabs	● After adjustment for age, sex, and antidepressant use, methylation of CpG unit 3.4.5. of exon I and CpG-3 of promoter IV remained significantly higher in depression.
Kang et al., 2013 [54]	CpG-rich region of the promoter between -694 and -577 relative to transcriptional start site, including 7 CpG sites.	Measured and averaged methylation at 7 CpG sites of *BDNF* promoter before 12 week antidepressant treatment (SSRIs, bupropion, mirtazapine, venlafaxine, amitriptyline, or imipramine) in Korean MDD patients. Assessed suicidal ideation during the treatment period.	PBMC	● Patients with lower *BDNF* promoter methylation showed greater improvement in BSS^3^ over the treatment period than patients with higher *BDNF* promoter methylation before antidepressant treatment.
Tadić et al., 2014 [55]	Twelve CpG sites within exon IV promoter	Measured *BDNF* methylation status in MDD patients before treatment with antidepressants and assessed outcomes at the study endpoint (ranging from 2–6 weeks) with HAM-D-21^4^.	PBMC	● Baseline methylation at CpG-87 predicted antidepressant response: Non-responders had a significantly lower methylated C-fraction than responders. ● Patients without any methylation at CpG-87 had higher risk of non-response to antidepressant than those with methylation. ● No significant effect of antidepressant class on the association between CpG-87 methylation and antidepressant response. ● DNA methylation of the 12 investigated CpG sites in exon IV did not change significantly during treatment from baseline to end point.
Kim et al., 2015 [57]	Nine CpG sites within exon VI promoter	Measured methylation of promoter of *BDNF* in peripheral blood of Korean ACS patients with and without any depressive disorder. They randomized 127 depressed participants to 24 weeks of escitalopram + ACS treatment and 128 to placebo + ACS treatment. The remaining 123 patients received standard medical treatment for ACS.	Peripheral blood leukocytes	● In the escitalopram-treated group, significantly higher average methylation % was found in participants with remission compared to those who did not remit. ● Persistence of baseline depressive disorder 1 year later was associated with a higher methylation at CpG-1 and higher average methylation % only in the placebo and the medical treatment-only groups, and not in the escitalopram group.
Chen et al., 2011 [58]	Exon IV promoter	Assessed relationship between *BDNF* methylation, MDD, antidepressant use, exon IV expression, and H3K27 tri-methylation in Caucasian male French Canadians post-mortem.	Prefrontal cortex (post-mortem)	● Antidepressant use was associated with significantly lower H3K27 methylation levels in *BDNF* exon IV promoter than the MDD without antidepressant and control groups.
Lopez et al., 2013 [59]	Exon IV promoter	Measured *BDNF* H3K27 trimethylation levels with ChIP^5^ and peripheral *BDNF* mRNA^6^ at baseline and after 8 weeks of citalopram. Subjects were divided into responders and non-responders based on final HAM-D score.	Peripheral blood	● Trimethylation of H3K27 at *BDNF* exon IV was significantly decreased after 8 weeks of citalopram in responders to citalopram, but not in non-responders. ● Significant negative correlation between change in depression severity and change in trimethylated H3K27 expression.

^1^ RT-PCR, reverse transcription polymerase chain reaction; ^2^ LES, Life Event Score; ^3^ Beck Scale for Suicide Ideation; ^4^ HAM-D-21, Hamilton Depression Rating Scale-21 items; ^5^ ChIP, chromatin immunoprecipitation; ^6^ mRNA, messenger RNA.

**Table 2:** Summaries of studies exploring relationship of antidepressants and *MAOA* DNA methylation.

Reference	Genomic Region Studied	Study Description	Tissue Examined	Findings
Checknita et al., 2018 [64]	Exon I promoter	*MAOA* was genotyped and methylation was measured via bisulfite conversion with PCR in Swedish women with and without substance use disorders, comparing those with and without a history of childhood sexual and/or physical abuse.	Genomic DNA extracted from saliva	● Among women with current depression, higher methylation was associated with past or current use of any medication (stimulants, hypnotics, anxiolytics, antidepressants, or antipsychotics) at CpG 7/8. ● When antidepressants were considered alone, no differences in methylation were found.
Domschke et al., 2015 [65]	Forty-three CpG sites within exon I promoter.	Methylation of *MAOA* was measured in peripheral blood of German MDD patients. Clinical response to 6 weeks of escitalopram was assessed by intra-individual changes in HAM-D-21 scores between weeks 1 and 6.	Peripheral blood	● In females, overall methylation across all 3 amplicons and single CpGs showed no association with intake of medication (SSRI, SSRI + mirtazapine, antipsychotics, or mood stabilizers). ● Average methylation across all CpGs showed no association with response to escitalopram after 6 weeks in females. ● Lower methylation at CpG-1 in Amplicon A and CpG-5 in Amplicon B were nominally associated with worse treatment response after 6 weeks of escitalopram in females. ● In males, neither average methylation across all sites nor methylation status of individual CpG sites showed association with response to escitalopram after 6 weeks.

**Table 3:** Summaries of studies exploring relationship of antidepressants and DNA methylation of serotonin and norepinephrine transporter genes.

Reference	Genomic Region Studied	Study Description	Tissue Examined	Findings
Booij et al., 2015 [67]	*SLC6A4* CpG sites 5–15 within the 214–625 bp regulatory region upstream of the promoter.	Analysis of *SLC6A4* methylation via pyrosequencing and luciferase reporter vector; compared with *SLC6A4* mRNA expression in T cells via RT-PCR; compared between healthy controls, childhood trauma, and MDD.	Peripheral blood T cells and monocytes	● SSRIs associated with increased methylation at CpG-11 and -12 compared to no antidepressants or dual-acting antidepressants. ● SSRI use predicted increased methylation at CpG-11 and -12.
Kang et al., 2013 [68]	*SLC6A4* CpG-rich region of the promoter between -479 and -350 relative to the transcriptional start site, including 7 CpG sites.	Measured DNA methylation of *SLC6A4* in Korean MDD patients. Patients were treated with a variety of antidepressants for 12 weeks and clinical outcome was measured by scales for depression, anxiety, functioning, disability, and quality of life before and after 12 weeks of antidepressant treatment.	Peripheral blood	● Higher methylation at CpG-2 and higher average *SLC6A4* methylation predicted less HAM-D improvement in depression. ● Higher methylation percentage at CpG-1 was associated with less improvement in HAM-A ^1^. ● Higher average promoter methylation was associated with decreased SOFAS ^2^. ● All above findings lost significance after Bonferroni correction.
Domschke et al., 2014 [69]	*SLC6A4* Nine CpG sites within the transcriptional control region upstream of exon 1A.	Analyzed blood sample DNA methylation status in Caucasian MDD patients. Clinical response to 6 weeks of escitalopram treatment was assessed by intra-individual changes in HAM-D-21 scores.	Peripheral blood	● Overall *SLC6A4* methylation in the analyzed amplicon showed no association with medication intake (SSRI vs. SSRI + mirtazapine), or comedication with antipsychotics or mood stabilizers. ● Average methylation across 9 CpG sites was significantly associated with response to escitalopram after 6 weeks: Lower methylation was associated with impaired treatment response, while higher methylation was associated with better treatment response. ● Average methylation across CpGs post-treatment showed a nominally significant association between lower methylation status and impaired treatment response. ● Methylation status of individual CpG-1 and -2 were significantly associated with treatment response after 6 weeks. CpG-4 methylation was nominally associated with treatment response.
Iga, et al., 2016 [70]	*SLC6A4* One CpG-rich region in the promoter, including 9 CpG sites.	Measured DNA methylation in peripheral blood of Japanese MDD patients before and after 8 weeks of treatment with various antidepressants. Clinical outcome was assessed with HAM-D.	Peripheral blood	Lower CpG-2 methylation levels were associated with greater clinical improvement as assessed by HAM-D scores.
Okada et al., 2014 [71]	*SLC6A4* CpG island in exon I promoter —sequence chr 17: 28562388–28563186	Measured DNA methylation and responses to antidepressant therapy in unmedicated, Japanese MDD patients.	Peripheral blood	● A significant increase in methylation was found in CpG-3 after 6 weeks of antidepressant treatment in MDD. ● Pre-treatment methylation of CpG-3 showed significant positive correlation with IR ^3^ in MDD. ● No significant difference in methylation rates between the patients with >50% IR and <50% IR. ● No correlation between IR and methylation change of CpG-3 before and after antidepressant treatment.
Bayles et al., 2013 [72]	*SLC6A2* Promoter region 1 (−515 bp to −225 bp), promoter region 2 (−180 bp–+167 bp), region A, and region B.	Study of *SLC6A2* methylation using EpiTYPER assays in an Australian population of MDD, panic disorder, and healthy controls. Subset comparison of *SLC6A2* promoter methylation before and after 3 month treatment with SSRIs in MDD and panic disorder.	Peripheral blood leukocytes	Statistically significant increase in methylation of CpG sites 14 and 15 (Region A) in MDD and panic disorder patients after 3 months of SSRI treatment.

^1^ HAM-A, Hamilton Anxiety Rating Scale; ^2^ SOFAS, Social and Occupational Functioning Assessment Score; ^3^ IR, improvement ratio.

**Table 4:** Summaries of studies exploring relationship of antidepressants and DNA methylation of serotonin receptor genes.

Reference	Genomic Region Studied	Study Description	Tissue Examined	Findings
Wang et al., 2018 [77]	*HTR1A* and *HTR1B* Ninety-six CpG sites within promoter regions.	Measured DNA methylation via PCR amplification of bisulfate-converted DNA, in Han Chinese MDD patients before and after 8 week treatment with escitalopram 10–20 mg daily.	Whole blood genomic DNA isolate	● Average methylation level of *HTR1A* or *HTR1B* was not significantly associated with treatment response to escitalopram. ● 2 CpG sites significantly predicted antidepressant response: CpG-668, amplicon *HTR1A*_1 and CpG-1401, amplicon *HTR1B*_4. Lower methylation at those sites was associated with impaired response to escitalopram. ● Patients with lower LES and higher DNA methylation at 4 CpG sites (*HTR1A*_1 CpG-659, *HTR1A*_1 CpG-668, *HTR1A*_1 CpG-706, *HTR1B*_2 CpG-107) responded better to escitalopram than those with higher LES and lower methylation. ● No significant difference in average DNA methylation of *HTR1A* and *HTR1B* between baseline and treatment week 8. ● Methylation at 4 individual CpG sites within *HTR1A/1B* was significantly increased after 8 weeks of escitalopram (*HTR1B*_1 CpG-336, *HTR1B*_2 CpG-105, *HTR1B*_2 CpG-107, *HTR1B*_4 CpG-1443). ● Significant differences in 6 CpG sites’ methylation in remitter and non-remitter groups (*HTR1A*_2 CpG-2793, *HTR1A*_2 CpG-2834, *HTR1A*_2 CpG-2927, *HTR1A*_2 CpG-2937, *HTR1B*_2 CpG-100, *HTR1B*_4 CpG-1401). ● DNA methylation was increased after escitalopram treatment in the remitter group, while there was no influence of escitalopram on methylation in the non-remitter group.
Gassó et al., 2017 [78]	*HTR1B* CpG islands in promoter, including 7 CpG sites. Chromosome 6: (77463994 – 77464019)	Measured DNA methylation after Spanish children with MDD, OCD^1^, or GAD^2^ completed 12 weeks of fluoxetine treatment for the first time and assessed whether DNA methylation was associated with clinical response to fluoxetine.	Peripheral blood	Negative correlation between average DNA methylation of the 7 CpGs analyzed in the *HTR1B* promoter and clinical response to fluoxetine as measured by GAF^3^/CGAS^4^.

^1^ OCD, obsessive compulsive disorder; ^2^ GAD, generalized anxiety disorder; ^3^ GAF, Global Assessment of Functioning Scale; ^4^ CGAS, Children’s Global Assessment Scale.

**Table 5:** Summaries of studies exploring relationship of antidepressants and DNA methylation of cytokine genes.

Reference	Genomic Region Studied	Study Description	Tissue Examined	Findings
Ryan et al., 2017 [82]	*IL6* 234 bp region of the promoter	Measured DNA methylation of *IL6* in peripheral tissue of French patients >65-years-old with and without depression, and with and without antidepressant treatment.	Buccal swabs	● Depression was associated with a 2.4% decreased overall *IL6* methylation compared to controls. ● Antidepressant use was associated with a mean 4.6% increase in methylation of *IL6*.
Powell et al., 2013 [85]	*IL11* CpG island in the promoter (chr 19: 55880511–55880989)	Measured baseline DNA methylation the *IL11* promoter in peripheral blood of Caucasian European MDD patients randomized to 12 weeks of either escitalopram or nortriptyline.	Whole blood genomic DNA isolate	● Methylation of CpG-5 predicted response to either antidepressant. ● Lower baseline CpG-5 methylation was associated with better antidepressant response. ● CpG-4 methylation predicted differential response to the two medications: High methylation levels were associated with better response to escitalopram, but with worse response to nortriptyline. ● Methylation at CpG-11 and rs1126757 significantly predicted response to treatment: Homozygous G allele (GG) individuals who had higher levels of CpG-11 methylation responded better to antidepressant treatment than those who were homozygous for the A allele (AA).

**Table 6:** Summaries of studies exploring relationship of antidepressants and whole genome DNA methylation.

Reference	Genomic Region Studied	Study Description	Tissue Examined	Findings
Takeuchi et al., 2017 [86]	Whole genome	Measured genome-wide methylation in peripheral blood cells of MDD patients before 6 week treatment with paroxetine. Compared the patients who were the best and worst responders to paroxetine.	Whole blood genomic DNA isolate	● 623 CpG sites had a >10% difference in methylation status between the best (BR) and worst (WR) responders to paroxetine, with 218 sites nominally significantly different and 2 sites significantly different: cg00594917 (*PPFIA4* exon I) and cg07260927 (in the 5′ UTR of *HS3ST1*). ● Methylation difference between WR and BR was greatest at cg00594917 in *PPFIA4* exon I. ● Hierarchical cluster analysis of 23 CpG sites in *PPFIA4* distinguished BR and WR patients except for 1 patient. ● Methylation of 6 CpG sites within *PPFIA4* was significantly different between BR and WR. At all 6 sites, WR had higher methylation than BR. ● Hierarchical cluster analysis of 28 CpG sites in *HS3ST1* distinguished BR and WR patients except for 1 patient. ● Methylation of 5 CpG sites within *HS3ST1* was significantly different between WR and BR. At 4 of 5 sites, methylation levels of WR were higher than those of BR.
Verhoeven et al., 2018 [88]	Whole genome	Applied Horvath’s epigenetic clock algorithm to calculate epigenetic age of leukocyte genomes of combat-exposed veterans with and without PTSD.	PBMC	● Current antidepressant use was associated with lower epigenetic age in veterans across the sample compared to subjects not taking antidepressants. ● PTSD subjects taking antidepressants had a significantly lower epigenetic age than participants without PTSD.

## Abbreviations

WHO	World Health Organization
MDD	Major depressive disorder
CpG	Cytosine-phosphate-guanine dinucleotides
BDNF	Brain-derived neurotrophic factor
PBMC	Peripheral blood mononuclear cells
CNS	Central nervous system
BD1	Bipolar disorder type 1
BD2	Bipolar disorder type 2
HC	Healthy controls
SSRI	Selective serotonin reuptake inhibitor
SNRI	Selective norepinephrine reuptake inhibitor
TCA	Tricyclic antidepressant
BD	Bipolar disorder
SI	Suicidal ideation
MAO-I	Monoamine-oxidase inhibitor
ACS	Acute coronary syndrome
H3K27	Histone H3, lysine 27
HAM-D	Hamilton Depression Rating Scale
MAO-A	Monoamine oxidase A
*SLC6A4*	Serotonin transporter
IR	Improvement ratio
*SLC6A2*	Sodium:norepinephrine symporter
*HTR1A*	5-hydroxytryptamine transporter 1A
*HTR1B*	5-hydroxytryptamine transporter 1B
LES	Life Event Score
*IL6*	Interleukin-6
*IL-11*	Interleukin-11
GENDEP	Genome-based Therapeutic Drugs for Depression Project
MADRS	Montgomery-Asberg Depression Rating Scale
BR	Best response
WR	Worst response
HS3ST1	Heparan sulfate-glucosamine 3-sulfotransferase 1
PTSD	Post-traumatic stress disorder
MECP2	Methyl-CpG-binding protein 2
TNF-α	Tumor necrosis factor alpha
PRISMA	Preferred Reporting Items for Systematic Reviews and Meta-Analyses
